# I just did it for the kids: mothering in the context of living with an increased risk of ovarian cancer

**DOI:** 10.1186/1897-4287-10-S2-A4

**Published:** 2012-04-12

**Authors:** A McEwen

**Affiliations:** 1 Central and Southern Regional Genetic Service, Wellington, New Zealand; 2 University of Otago, New Zealand

## 

Hereditary breast and ovarian cancer syndromes are rare genetic disorders conferring a significant lifetime risk of developing breast and/or ovarian cancer. Women with a strong family history of breast and ovarian cancer face decisions regarding genetic testing, cancer surveillance and risk reducing surgery.

This paper draws on interview data from a group of thirty-two Pakeha New Zealand women living with an increased risk of breast and ovarian cancer.

“Getting on with it” emerged as a dominant theme, as the way in which most of these women approached their risk. “Getting on with it” appears to be a deeply entrenched social, cultural and gendered expectation in New Zealand, perhaps influenced by our history as a settler society and the more recent influences of neo-liberal governance. This approach to risk is influential in guiding the management decisions that these women are making.

Mothering is central to the identity of many of the participants in this study. They mother their children within the context of the increased cancer risk. These women identify a strongly felt responsibility to be there to care for their children. Alongside a strongly voiced desire to “get on with it”, they use their role as mothers to motivate their decisions regarding risk reducing surgery. I argue that choosing to undergo the removal of healthy body parts in order to reduce risk and remain alive to fulfil role expectations provides a symbolic and gendered representation of women as carers and nurturers.

